# A Vision-Based Sensing Framework for PPE Detection and Safety Harness Compliance Recognition in High-Formwork Construction Environments Using YOLO-ILB

**DOI:** 10.3390/s26134147

**Published:** 2026-07-01

**Authors:** Gang Yao, Lang Liu, Yang Yang, Xiaodong Cai

**Affiliations:** 1 School of Civil Engineering, Chongqing University, Chongqing 400045, China; yaogang@cqu.edu.cn (G.Y.); 202416131378@stu.cqu.edu.cn (L.L.); 202316131156t@stu.cqu.edu.cn (X.C.); 2 State Key Laboratory of Safety and Resilience of Civil Engineering in Mountain Area, Chongqing 400045, China

**Keywords:** high-formwork support system, personal protective equipment detection, safety harness compliance, construction safety, small object detection

## Abstract

Automated vision-based sensing for personal protective equipment (PPE) compliance in high-formwork support system (HFSS) construction environments faces three compounding challenges: extreme within-image scale variation, dense scaffold occlusions, and small safety hook targets prone to missed detection. Existing sensing systems address only binary presence detection and cannot assess whether safety harnesses are anchored in compliance with regulatory requirements. This paper proposes YOLO-ILB, a lightweight task-specific object detector built on YOLO11n with three targeted improvements. The C3k2_IDWC module replaces standard convolutions with multi-branch Inception depthwise convolutions, improving multi-scale feature discrimination at reduced computational cost. The SPPF_LSKA module embeds large separable kernel attention into the SPPF aggregation path, strengthening global context awareness to suppress scaffold background interference. A BiFPN neck replaces the original PAN structure, enabling bidirectional cross-scale weighted feature fusion to balance detection of small hooks and large harnesses within a sin gle image. A UAV-based sensing dataset was constructed using a DJI Mini 3 Pro (4032 × 3024 px) across 17 real construction sites under varied altitudes, viewing angles, and illumination conditions, yielding 2700 annotated images across five object categories. YOLO-ILB achieves mAP50 = 0.939 with only 1.923 M parameters and 5.7 G FLOPs at 262.3 FPS, outperforming eight mainstream YOLO baselines while remaining deployable on resource-constrained edge computing nodes. A geometry-based compliance algorithm further classifies three harness anchoring states—correct high anchoring, incorrect low anchoring, and unclipped or excessively distant hook—without additional sensors or annotations, achieving 90.82% overall accuracy on 305 field instances and extending the sensing system from presence detection to regulatory compliance assessment.

## 1. Introduction

Large Separable Kernel Attention (LSKA) falls from height are the leading cause of fatal injuries in the construction industry worldwide. In the United States, falls, slips, and trips caused 421 construction fatalities in 2023, representing 39.2% of all industry deaths [1]. HFSS, widely used in large-span and high-rise projects, are among the most hazardous work environments. Their structural complexity, considerable working heights, and concurrent multi-trade operations amplify fall risk substantially [2]. Workers on HFSS are required to wear full-body harnesses and attach safety hooks to compliant anchor points. Chinese national standards stipulate that the anchor point must be located above the worker, limiting free-fall distance to 2 m or less [3]. Violations of this high-anchoring requirement significantly worsen injury outcomes. Manual inspection alone cannot enforce compliance reliably, as it is constrained by limited inspector access, inherent subjectivity, and the inability to monitor continuously [4].

Deep learning-based computer vision has opened practical avenues for automated PPE compliance monitoring on construction sites. YOLO-family detectors, in particular, have demonstrated strong real-time performance in detecting helmets [5,6] and safety vests [7]. Subsequent work has extended these methods to harness detection using task-optimized architectures [8]. Nevertheless, nearly all existing studies treat detection as a binary problem, reporting only whether PPE is present rather than whether it is used correctly [9]. For safety harnesses, this distinction is critical. A harness provides meaningful fall protection only when the anchor point is positioned correctly relative to the worker.

Drones have become an effective data acquisition platform for HFSS monitoring. Their ability to navigate confined scaffold spaces and capture imagery from multiple angles and altitudes compensates for the coverage limitations of fixed cameras. Prior work has demonstrated that drone-mounted systems can detect safety violations in real time during elevated work. However, these systems remain focused on presence detection and have not addressed anchor point compliance.

Detection in HFSS environments presents challenges that go beyond those of general construction sites. Safety hooks are small targets that share color and texture characteristics with scaffold connectors, making them prone to missed detection. Helmets, harnesses, and hooks span an order of magnitude in pixel area within a single image. Dense scaffold members cause complex occlusions that degrade feature quality. Standard YOLO architectures handle general scenes effectively but have not been optimized for this combination of challenges. To address these shortcomings, this paper makes the following contributions:

(1) YOLO-ILB is proposed, a PPE detector for HFSS built on YOLO11n. Three targeted improvements are integrated: C3k2_IDWC for enhanced multi-scale feature extraction with reduced computational cost; SPPF_LSKA for strengthened global context awareness at the backbone tail; and a BiFPN neck for bidirectional cross-scale weighted feature fusion.
(2) A dedicated HFSS PPE dataset is constructed from 2700 drone-captured images collected across 17 real construction sites and multiple project types. Five categories are annotated, covering both the presence and absence of each PPE item.
(3) A geometry-constrained harness compliance detection method is proposed. It classifies three harness usage states: correct high-point anchoring, incorrect low-point anchoring, and dangerous unclipped or excessively distant hook. The method achieves 90.82% overall accuracy on 305 field instances without additional sensors or annotations.

## 2. Literature Review

Computer vision-based PPE detection has attracted sustained research attention over the past decade. Early approaches relied on traditional image processing and hand-crafted features. The introduction of convolutional neural networks (CNNs) and anchor-based detectors such as Faster R-CNN and SSD substantially improved detection accuracy for helmets and safety vests [6]. The YOLO family subsequently became the dominant framework for real-time construction site PPE detection, integrating classification and localization into a single forward pass. Comparative evaluations on construction-specific datasets have shown that YOLOv5x achieves the highest mean average precision in multi-class PPE detection. Successive versions from YOLOv8 through YOLOv10 to YOLO11 have progressively refined backbone feature extraction, label assignment strategies, and neck fusion architectures, further improving the accuracy-efficiency trade-off [10]. Recent work has applied YOLOv10 with a Swin Transformer backbone to construction site PPE detection, achieving AP50 scores above 88% across multiple PPE categories on benchmark datasets [11].

Despite these advances, most PPE detection research addresses only equipment presence [6,12]. Studies on helmet detection, safety vest detection [7], and comprehensive PPE compliance monitoring have primarily produced binary labels indicating whether an item is worn, without assessing whether it is configured or used correctly [5]. Existing methods also face challenges in detecting small and occluded objects, particularly in cluttered industrial backgrounds. These limitations are especially consequential in HFSS scenarios, where the spatial configuration of the harness relative to the anchor point is the primary determinant of protective effectiveness.

Safety harness detection has received comparatively less attention than helmet or vest detection, reflecting both its greater visual complexity and the historical focus of monitoring efforts on presence-based detection. Wu et al. proposed a computer vision framework for harness detection on construction sites, demonstrating the feasibility of vision-based approaches for identifying harness violations. Subsequent studies extended these methods using attention-based single-stage detectors and custom loss functions to improve performance in elevated work scenarios [8]. Lim et al. systematically analyzed South Korean regulatory requirements for safety harnesses and hooks on scaffold systems, identifying the visual recognition elements corresponding to harness wearing, hook connection, and anchor facility installation [13].

None of these studies, however, distinguish between a correctly positioned anchor point and an incorrectly positioned one. The specific question of high-anchoring compliance, namely verifying whether the safety hook is anchored above the worker’s dorsal attachment point, has received little direct attention in the literature [9,13]. This gap is significant. A worker may wear a harness correctly yet anchor it at a position that violates fall protection standards, substantially reducing protection in the event of a fall.

Drones have emerged as an effective platform for construction site safety monitoring, overcoming the spatial limitations of ground-based inspection. Research combining drone systems with deep learning has demonstrated the feasibility of real-time safety activity monitoring from drone-captured imagery. Such systems have reliably detected safety violations during elevated work, highlighting the complementary role of aerial perspectives in identifying hazards that are difficult to observe from the ground. In HFSS contexts specifically, where workers operate at height within three-dimensionally distributed scaffold structures, drones are particularly suited for data collection because fixed cameras cannot adequately cover all working levels and orientations.

Detecting small and occluded objects, such as safety hooks against complex scaffold backgrounds, remains a core challenge for CNN-based detectors. Feature Pyramid Networks (FPNs) [14] address scale variation by propagating high-level semantic information to lower-level feature maps through a top-down pathway. Path Aggregation Networks (PANs) [15] complement this with a bottom-up pathway to strengthen the propagation of shallow features. Bi-directional Feature Pyramid Network (BiFPN) [16] extends both architectures by introducing bidirectional cross-scale connections with learnable input fusion weights, enabling adaptive weighting of contributions from different feature scales. These multi-scale fusion strategies are especially relevant for HFSS PPE detection, where helmets, harnesses, and hooks may differ by an order of magnitude in pixel area within a single image.

The review above identifies three challenges that remain inadequately addressed and that motivate the present study. First, no PPE detection model has been specifically optimized for HFSS, where small safety hooks, scaffold background clutter, and extreme within-image scale variation collectively degrade detection performance. Second, existing harness detection systems evaluate only wearing status and cannot assess regulatory compliance of anchor point positioning. Third, although drone imagery has been applied to general construction safety monitoring, no reported system combines it with an anchor point compliance algorithm. This paper addresses all three gaps through the YOLO-ILB model and the geometry-constrained compliance recognition method.

## 3. Methods

### 3.1. Dataset Construction

Workers on HFSS typically operate at considerable heights above ground level, with limited space available for movement at the base of the structure. Handheld image acquisition under these conditions is both operationally inconvenient and inefficient, and cannot satisfy multi-scale imaging requirements. A DJI Mini 3 Pro drone (Shenzhen Da-Jiang Innovation Technology Co., Ltd., Shenzhen, China) was adopted as the primary data acquisition platform. Its compact form factor allows it to navigate gaps within the scaffold structure.

**Figure 1:**
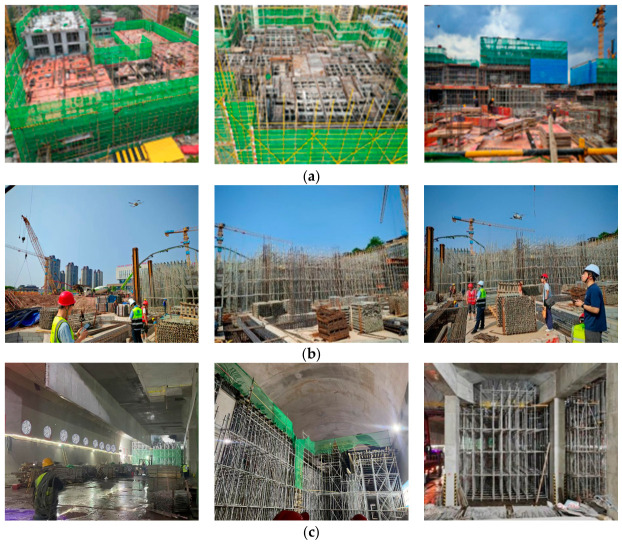
The Construction Sites for Safety Equipment Image Data Collection. (**a**) Example Images for Data Collection at Residential Construction Sites. (**b**) Example Images for Data Collection at Ground Parking Lot. (**c**) Example Images for Data Collection at Subway Station.

Although large public datasets are available for general computer vision research, no dedicated dataset exists for safety equipment worn by HFSS workers. Data were therefore collected through two complementary channels. The first channel involved controlled experimental collection. Typical HFSS construction scenarios were reproduced on a test scaffold, and workers were asked to demonstrate various states of safety equipment wearing and usage. Drone imagery was then captured at multiple scales. Experimental collection offers advantages in data volume and allows precise control over the balance of samples across different equipment states. Indoor lighting can also be adjusted to simulate varied outdoor illumination conditions, enhancing scene diversity. The second channel involved on-site collection at active construction projects. Images were captured across multiple sites in Chongqing, covering residential buildings, underground metro stations, sports venues, and elevated bridges. Cross-project collection enriches scene diversity and supports model generalization. Field-collected data also reflect actual construction conditions more faithfully than laboratory simulations. The two collection channels are mutually complementary: controlled experimental collection prioritizes sufficient coverage of rare equipment states (e.g., low-anchoring and no-harness conditions), while real construction-site collection prioritizes scene diversity and generalization, together ensuring dataset representativeness across both category states and environmental conditions. Representative collection sites are shown in Figure 1.

Images were captured using the DJI Mini 3 Pro with an aspect ratio of 4:3 and a resolution of 4032 × 3024 pixels. The key technical specifications of the UAV platform are summarized in Table 1. Data collection was conducted between April 2024 and August 2025 across 17 construction projects, yielding a total of 2700 high-resolution images. The dataset was split into training, validation, and test sets at a ratio of 6:3:1, with stratified sampling applied across categories to ensure balanced distribution. Among the 2700 collected images, a total of 25,776 annotated instances were labeled across the five categories. The distribution per category is as follows: helmet (7192), no-helmet (4511), harness (6212), no-harness (3866), and safety hook (3995), reflecting the natural imbalance between correctly worn and missing equipment in real construction scenarios. Regarding data source composition, 1257 images were acquired under controlled experimental conditions using a test scaffold with adjustable indoor lighting to simulate various outdoor illumination levels, while the remaining 1443 images were captured from 17 active construction sites across Chongqing, covering residential buildings, underground metro stations, sports venues, and elevated bridges. This split ensures both balanced representation of rare equipment states (e.g., no-harness) and high ecological validity from field deployments.

### 3.2. YOLO-ILB Model Architecture

Selecting a baseline model for safety equipment detection requires balancing detection performance, computational efficiency, parameter overhead, and deployment feasibility. HFSS environments present compounding difficulties: dense scaffold members create mutual occlusions among components, and helmets, harnesses, and hooks exhibit pronounced scale differences within a single image. These differences are further influenced by shooting distance, viewing angle, and lighting conditions. A suitable detection model must therefore handle multi-scale targets effectively, adapt to environmental variation, and maintain a practical balance between accuracy and inference speed. The architectural features and key optimizations of YOLOv8, YOLOv10, YOLO11, and YOLO12 were analyzed and are summarized in Table 2.

Across the four versions, architectural continuity is strong, with improvements concentrated in internal modules built progressively on the YOLOv5 framework. From YOLOv10 onward, dual-label assignment replaces traditional non-maximum suppression, improving detection quality. Although YOLO12 offers higher accuracy than YOLO11, its greater reliance on attention mechanisms limits the inference efficiency characteristic of CNN-based architectures. YOLO12 also requires optimized attention computation libraries and high-performance GPUs to operate effectively, imposing stricter demands on compute resources and deployment environments. Given the emphasis on fast inference and high-resolution image processing in HFSS scenarios, YOLO11 was selected as the baseline model.

Three targeted improvements were integrated into the YOLO11n baseline to address the specific challenges of HFSS detection: large scale variation among safety equipment items, strong interference from scaffold backgrounds, and frequent missed detection of small targets.

(1) C3k2_IDWC: Multi-Scale Feature Extraction

The C3k2 module in YOLO11 extracts features through stacked Bottleneck structures. Standard large-kernel convolutions in this module incur high parameter counts and memory access overhead [17], and are insufficient for capturing fine-grained features of small targets. In HFSS environments, safety hooks closely resemble scaffold connectors in color and texture, making inadequate feature extraction a primary cause of missed detections.

**Figure 2:**
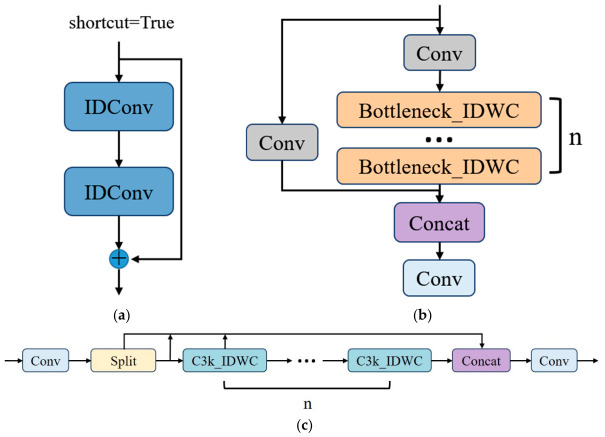
C3k2_IDWC Feature Extraction Module. (**a**) Bottleneck_IDWC. (**b**) C3k_IDWC. (**c**) C3k2_IDWC.

To address this, Inception Depthwise Convolution (IDWC) is embedded into the Bottleneck units of C3k2, replacing the original standard convolutions. The structures of Bottleneck_IDWC and C3k_IDWC are shown in Figure 2a and Figure 2b, respectively, and the resulting C3k2_IDWC module is shown in Figure 2c. The module reduces parameter count and computational complexity without materially affecting detection performance, improving deployment suitability on resource-constrained edge devices at construction sites.

In IDWC, the input feature *X* is split into four parts and processed in parallel by separate convolution branches, and then concatenated along the channel dimension to strengthen inter-branch feature interaction. The computation proceeds as follows:

(1) $$X_{\mathrm{hw}},X_{w},X_{h},X_{\mathrm{id}}=\mathrm{Split}(X)=X_{:,:g},X_{:g:2g},X_{:2g:3g},X_{:3g:}$$

(2) $$X_{\mathrm{hw}}^{\prime }={\mathrm{DWConv}}_{k_{s}\times k_{s}}^{g\to g}g(X_{\mathrm{hw}})$$

(3) $$X_{w}^{\prime }={\mathrm{DWConv}}_{l\times k_{b}}^{g\to g}g(X_{w})$$

(4) $$X_{h}^{\prime }={\mathrm{DWConv}}_{k_{b}\times l}^{g\to g}g(X_{h})$$

(5) $$X_{\mathrm{id}}^{\prime }=X_{\mathrm{id}}$$

(6) $$X^{\prime }=\mathrm{Concat}(X_{\mathrm{hw}}^{\prime },X_{w}^{\prime },X_{h}^{\prime },X_{\mathrm{id}}^{\prime })$$

where h and w denote the spatial height and width dimensions; *g* is the number of channels per branch; *k*_s_ and *k*_b_ are the square and asymmetric kernel sizes. Compared with standard large-kernel convolutions, IDWC maintains strong feature extraction capability and a large receptive field while processing input features across four parallel branches. This structure reduces computational overhead and memory access pressure, promotes inter-channel information flow, and balances feature representation quality against computational efficiency.

(2) SPPF_LSKA: Global Context Enhancement

The SPPF module in YOLO11 fuses features through multi-scale pooling alone, without modeling global context. High-level features at the backbone tail are semantically rich but spatially coarse, making them susceptible to background interference from scaffold members and debris. This interference leads to localization errors in safety equipment.

**Figure 3:**
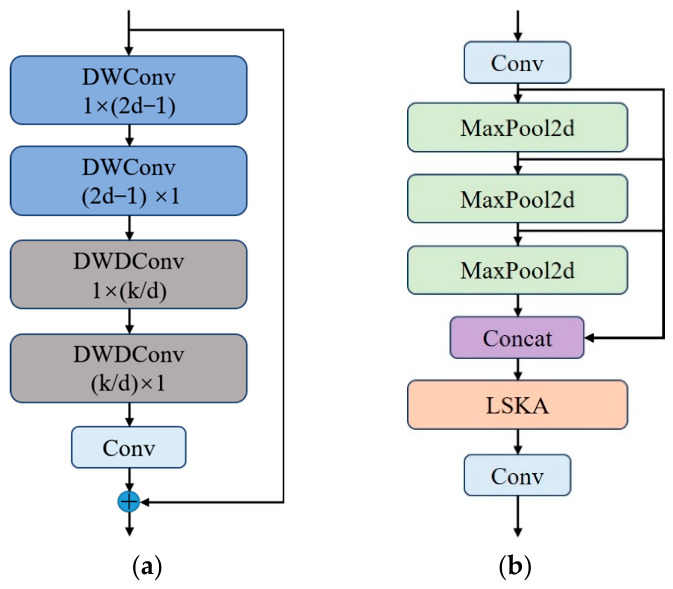
SPPF_LSKA Feature Enhancement Module. (**a**) LSKA. (**b**) SPPF_LSKA.

Large Separable Kernel Attention (LSKA) [18] is embedded into the SPPF feature aggregation path to address this limitation. LSKA first applies two depthwise separable convolution layers to extract horizontal and vertical feature information separately. A depthwise separable dilated convolution layer then expands the receptive field and enhances feature representation. A final convolution layer fuses the multi-branch features to produce an attention map, which is multiplied element-wise with the input features. This structure is shown in Figure 3a. The mechanism directs the network toward salient safety equipment features while suppressing background interference from the complex HFSS environment.

The LSKA computation is defined as:

(7) $$X^{C}=\sum_{H,W}W_{\left(2d-1\right)\times 1}^{C}*\left(\sum_{H,W}W_{1\times \left(2d-1\right)}^{C}*F^{C}\right)$$

(8) $$Y^{C}=\sum_{H,W}W_{\left|\frac{k}{d}\right|\times 1}^{C}*(\sum_{H,W}W_{1\times \left|\frac{k}{d}\right|}^{C}*X^{C})$$

(9) $$A^{C}=W_{1\times 1}*Y^{C},H^{C}=A^{C}⊗F^{C}$$

where *C* is the number of channels; *H* and *W* are the feature map height and width; *k* and *d* are the kernel size and dilation rate; *F^C^* is the input feature map. *X^C^* is obtained by applying horizontal convolution with kernel $W_{(2d-1)\times 1}^{C}$ followed by vertical convolution with kernel $W_{(2d-1)\times 1}^{C}$ on *F^C^*. *X^C^* is obtained by applying dilated convolution to *X^C^*. The final LSKA output *H^C^* is the Hadamard product of the attention map *A^C^* and the input feature map *F^C^*.

The SPPF_LSKA module is formed by embedding LSKA into the feature aggregation path of the original SPPF structure [19], as shown in Figure 3b. It retains the multi-scale pooling advantage of SPPF while compensating for the limited global modeling capacity of pooling alone through LSKA’s large-kernel decomposition and adaptive spatial weighting. The resulting structure enhances high-level semantic feature representation at low computational cost, better serving safety equipment recognition in HFSS scenarios.

(3) BiFPN: Bidirectional Weighted Feature Fusion

**Figure 4:**
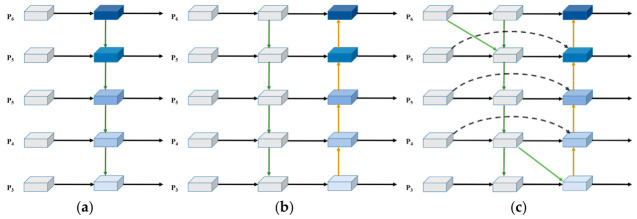
Multi-scale Feature Fusion Strategy. (**a**) FPN. (**b**) PAN. (**c**) BiFPN.

Although PAN introduces a bottom-up path to supplement FPN’s top-down pathway, it merges features at each scale with equal weights, without adaptive weighting to reflect the varying contributions of features from different scales. BiFPN addresses this by introducing learnable fusion weights. Small-target features at high resolution are easily overwhelmed by large-target semantic features at low resolution, and the additional pathways increase computational overhead. FPN and PAN are the two most widely adopted neck structures in mainstream object detectors; their topologies are illustrated in Figure 4a,b. FPN propagates deep semantic information into shallow feature maps through a top-down path, enriching low-level semantic representation. However, its unidirectional design limits the feedback of shallow detail into higher layers, leaving high-level features with insufficient spatial and structural awareness. PAN supplements this with a bottom-up path to reinforce shallow feature propagation upward. In practice, this additional pathway increases computational cost. Furthermore, direct feature merging without adaptive weighting can dilute important information and degrade final detection performance.

BiFPN addresses the limitations of both FPN and PAN by constructing a bidirectional fusion structure, as shown in Figure 4c. It removes single-input nodes that contribute marginally to fusion and establishes cross-layer connections between input and output nodes, reducing redundant computation while improving inter-level information exchange. To account for the varying contributions of features at different resolutions, BiFPN introduces learnable weights that adaptively scale the contribution of each input feature. The weighted feature fusion formula is:

(10) $$out=\sum_{i}\frac{w_{i}}{\varepsilon +\sum_{j}w_{j}}•I_{i}$$

where *w_i_* are the learned weights with $w_{i}\;\geq \;0$; ε=0.0001 is a numerical stability constant; and *I_i_* is the input feature of branch *i*. Introducing BiFPN into the neck network addresses the detection difficulties caused by scale variation among HFSS worker safety equipment. Its bidirectional cross-scale weighted fusion mechanism strengthens information exchange across feature levels, enabling precise localization of small targets while maintaining global awareness of large ones. The enhanced multi-scale features output by the improved neck improve detection performance in complex construction environments.

**Figure 5:**
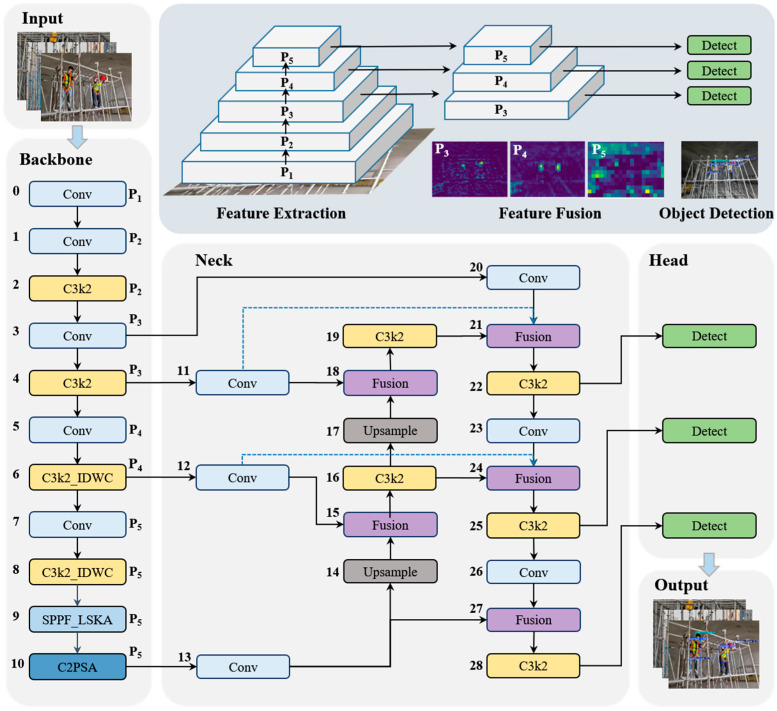
YOLO-ILB Model Architecture.

The final YOLO-ILB model integrates C3k2_IDWC, SPPF_LSKA, and BiFPN into the YOLO11n baseline through targeted structural modifications. The resulting model exhibits greater robustness to interference under complex construction conditions, adapts more effectively to varied scenes, and improves detection across safety equipment targets of different sizes. The YOLO-ILB architecture is shown in Figure 5.

### 3.3. Recognition of High-Anchoring and Low-Anchoring

YOLO-ILB provides efficient detection of helmets, harnesses, and hooks. Presence detection alone, however, is insufficient to ensure safety compliance on HFSS. Workers must not only wear a harness but also attach it correctly. National standards [3] require that the anchor point be located above the worker during elevated work, limiting free-fall distance to 2 m or less. On HFSS, low-anchoring positions typically fail to meet this requirement, increasing the risk of collision with scaffold members and loss of stability in a fall. Current computer vision applications in construction safety have largely focused on equipment presence and have not examined the state of safety hook attachment in depth [9,20]. A harness high-anchoring recognition method is therefore developed, using the relative spatial positions of the detected safety hook and harness to determine the anchoring state.

**Figure 6:**
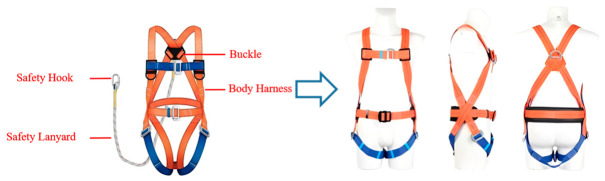
Five-point Safety Harness.

Work on HFSS constitutes typical elevated-height operation, requiring workers to wear fall-arrest full-body harnesses. This type of protective equipment follows a five-point full-body configuration, with standard components including the body harness, safety lanyard, safety hook, and connecting buckles, as shown in Figure 6. The body harness secures the torso and lower limbs, while the lanyard and hook together provide fall arrest. In correct use, the lanyard originates from the central dorsal attachment point located between the worker’s shoulder blades, which serves as the primary load-bearing point during a fall.

**Figure 7:**
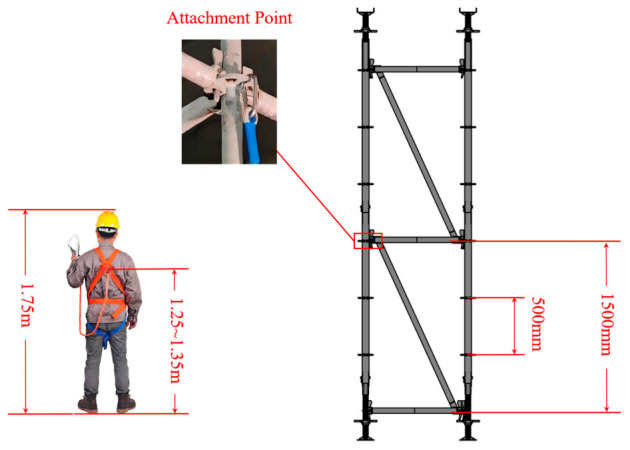
Dimensions of frame and operator.

Establishing the visual recognition logic requires analysis of both the physical geometry of the scaffold and human body proportions. In socket-and-spigot ringlock steel tube support systems, the most common step height in practice is 1.5 m, with one ringlock disc positioned every 0.5 m along each vertical post, giving a vertical spacing of 1.5 m between adjacent horizontal members. Workers typically attach their safety hook to the horizontal member or ringlock node at the top of the current working bay. Based on a reference adult male height of 1.75 m, the chest and inter-scapular height corresponds to approximately 70–75% of body height [21]. Accounting for the dorsal attachment point being slightly higher than the chest, the height of the dorsal attachment point above the floor is estimated to fall between 1.25 m and 1.35 m, as illustrated in Figure 7.

From this dimensional analysis, the physical height of the safety hook exceeds that of the worker’s dorsal attachment point and the overall harness centroid under compliant working conditions. To quantify this, a Cartesian coordinate system is established with the origin at the lower-left corner of the image. The height constraint is then mapped to a geometric position constraint: the vertical coordinate of the safety hook detection box center must be greater than the vertical coordinate of the harness detection box center, as expressed in Equation (11). This geometric constraint is consistent with the high-anchoring safety requirement.

Before comparing coordinates, the validity of detected targets must be verified. Given the physical length constraint of the safety lanyard, the worker and their associated safety hook must satisfy the geometric constraint in Equation (12). If this constraint is not satisfied, the target is classified as a background false detection or a hook not belonging to the current worker, and is excluded as invalid.

(11) $$y_{\mathrm{hook}}>y_{\mathrm{harness}}$$

(12) $$d\leq \lambda \cdot h_{\mathrm{harness}}$$

where *h_harness_* is the height of the harness detection box; *d* is the Euclidean distance between the centers of the safety hook and harness detection boxes; and *λ* is the lanyard length scaling factor. Based on the standard lanyard length of 1.5 m to 2.0 m and the proportional relationship between *h_harness_* and the worker’s physical torso height, the empirical range of *λ* is 2.5 to 3.5. A default value of *λ* = 3.0 was adopted in this study, consistent with the pixel scale of a standard 1.5 m lanyard at typical drone capture distances. In practical deployment, λ should be adjusted to match the specific lanyard specification in use.

**Figure 8:**
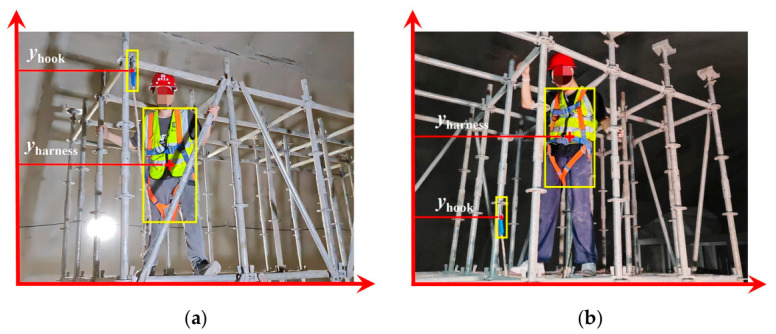
Single-target harness usage detection. (**a**) Compliant Harness Usage. (**b**) Non-Compliant Harness Usage.

For images containing a single worker that passes validity verification, harness usage state is determined as follows. If $y_{hook}>y_{harness}$, the harness is classified as compliant high anchoring. If $y_{hook}\leq y_{harness}$, it is classified as non-compliant low anchoring. Examples are shown in Figure 8.

HFSS construction frequently involves multiple workers operating within the same or adjacent bays. In such multi-target scenarios, images typically contain multiple harness and hook detection boxes. Single-target recognition logic does not apply directly. The hook-to-worker assignment problem must first be resolved, meaning that each detected hook must be correctly associated with its corresponding worker. The core of multi-target recognition therefore lies in establishing a robust association matching mechanism.

**Figure 9:**
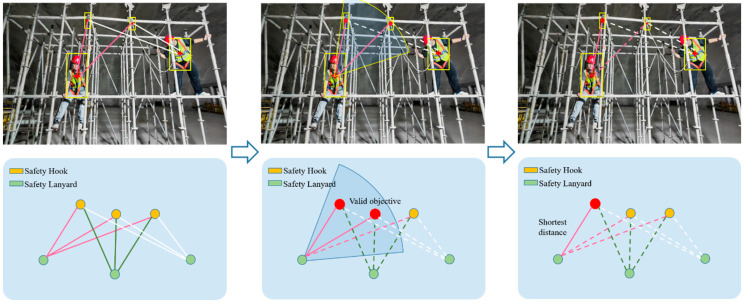
Safety hook matching process.

The hook matching procedure consists of three steps. First, all harness and hook detection boxes in the image are enumerated and the Euclidean distances *d_ij_* between their centers are computed, forming a distance association matrix *D*, as given in Equations (13) and (14). Second, the validity criterion from single-target recognition is applied to filter candidate hooks within the effective lanyard radius, discarding invalid targets, as expressed in Equation (15). Third, each harness is matched to a unique hook based on the minimum Euclidean distance principle, and matched hooks are marked to prevent duplicate assignment. The complete hook matching procedure is illustrated in Figure 9.

(13) $$d_{ij}=\sqrt{{\left(x_{\mathrm{ha},i}-x_{\mathrm{ho},j}\right)}^{2}+{\left(y_{\mathrm{ha},i}-y_{\mathrm{ho},j}\right)}^{2}},(i=1,\ldots ,M;j=1,\ldots ,N)$$

(14) $$D=\left[\begin{array}{cccc} d_{11} & d_{12} & \ldots & d_{1N}\\ d_{21} & d_{22} & \ldots & d_{2N}\\ \vdots & \vdots & \ddots & \vdots \\ d_{M1} & d_{M2} & \ldots & d_{MN} \end{array}\right]$$

(15) $$d_{ij}\leq \lambda \cdot h_{\mathrm{ha},i}$$

where *d_ij_* is the Euclidean distance between the center of the *i*-th harness detection box and the center of the *j*-th hook detection box; *x*_ha,I_, *y*_ha,j_ are the coordinates of the *i*-th harness box center; *x*_ho,*i*_, *y*_ho,*j*_ are the coordinates of the *j*-th hook box center; *M* is the total number of harness detection boxes; *N* is the total number of hook detection boxes; *y*_ho,*i*_ is the height of the *i*-th harness detection box; and λ is the lanyard length scaling factor.

By combining lanyard geometric constraints with a minimum-distance matching strategy, this multi-target mechanism reliably resolves hook-to-worker assignment ambiguities in multi-person HFSS scenarios, providing a robust algorithmic basis for harness high-anchoring state recognition.

## 4. Experiment

### 4.1. Comparative Experiment

**Figure 10:**
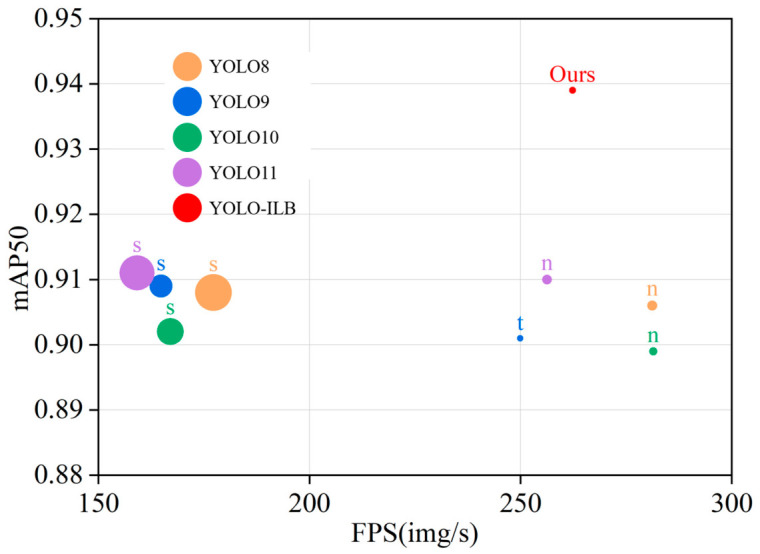
Comparison Results with Different Baseline Models.

To objectively evaluate the overall performance of YOLO-ILB on the HFSS safety equipment detection task and identify directions for further optimization, a comparative experiment was conducted against mainstream YOLO baseline models. All models were built on the Ultralytics 8.3.119 platform under a unified training environment and hyperparameter configuration, minimizing the influence of non-model factors on results. Each model was trained to full convergence of the loss function, ensuring that its performance was fully realized before comparison. Results are reported in Table 3 and Table 4. Each YOLO version was evaluated using its two officially released model-size variants (a smaller n/t variant and a larger s variant) to capture the full accuracy–efficiency range achievable by that baseline family, rather than a single fixed configuration. These results are visualized in Figure 10, where the horizontal axis denotes inference speed (FPS), the vertical axis denotes mAP50, and the diameter of each marker is proportional to the model’s parameter count (M); no trend line is drawn, as each point represents an independent model configuration rather than a sample along a continuous relationship.

The results demonstrate that YOLO-ILB achieves a Pareto-optimal balance among accuracy, model size, and inference speed for HFSS safety equipment detection. Its mAP50 of 0.939 exceeds the second-best baseline, YOLO11s, by 2.8 percentage points and surpasses the native YOLO11n by 3.2% relatively, confirming the synergistic benefit of the three proposed modules for small-target hook detection and feature fusion in complex scenes. In terms of compactness, YOLO-ILB contains only 1.923 M parameters, equivalent to 20.4% of YOLO11s. Although its parameter count is marginally higher than that of the most compact baseline, YOLOv9t (1.731 M), YOLO-ILB exceeds it in mAP50 by 3.8 percentage points while reducing FLOPs to 5.7 G, representing a clear advantage for deployment on resource-constrained edge devices at construction sites.

Regarding inference speed, YOLO-ILB runs at 262.3 FPS, substantially faster than YOLO11s (159.2 FPS) and closely matching the baseline YOLO11n (256.3 FPS). It falls slightly below the speed-oriented YOLOv10n (281.4 FPS) but surpasses it in mAP50 by 4.0 percentage points. These results confirm that the proposed improvements do not sacrifice real-time capability. The model fully meets the millisecond-level response requirements of drone-based HFSS inspection and provides a deployable solution for automated safety monitoring at construction sites.

Per-category detection results are summarized in Table 4. Helmets and harnesses, as the two largest equipment categories in pixel area, achieve AP50 values of 0.975 and 0.968, respectively, with precision above 0.956 and recall above 0.950, confirming that these targets are reliably detected across all scenes and drone positions. The absence categories, no-helmet (0.949) and no-harness (0.952), also perform well, demonstrating that the model successfully learns contextual cues for distinguishing compliant from non-compliant workers. Safety hooks yield the lowest AP50 of 0.851, with a recall of 0.846 that is noticeably lower than precision (0.884), indicating that missed detections rather than false positives constitute the primary source of error. This asymmetry is consistent with the nature of the target: safety hooks are physically small, share color and texture characteristics with scaffold connectors, and can be partially occluded by scaffold members, all of which reduce the model’s ability to consistently delineate the hook boundary against complex backgrounds. Nevertheless, the overall mAP50 of 0.939 demonstrates that the three proposed modules—C3k2_IDWC, SPPF_LSKA, and BiFPN—collectively raise detection performance across all five categories to a level sufficient for reliable safety monitoring in HFSS environments.

**Figure 11:**
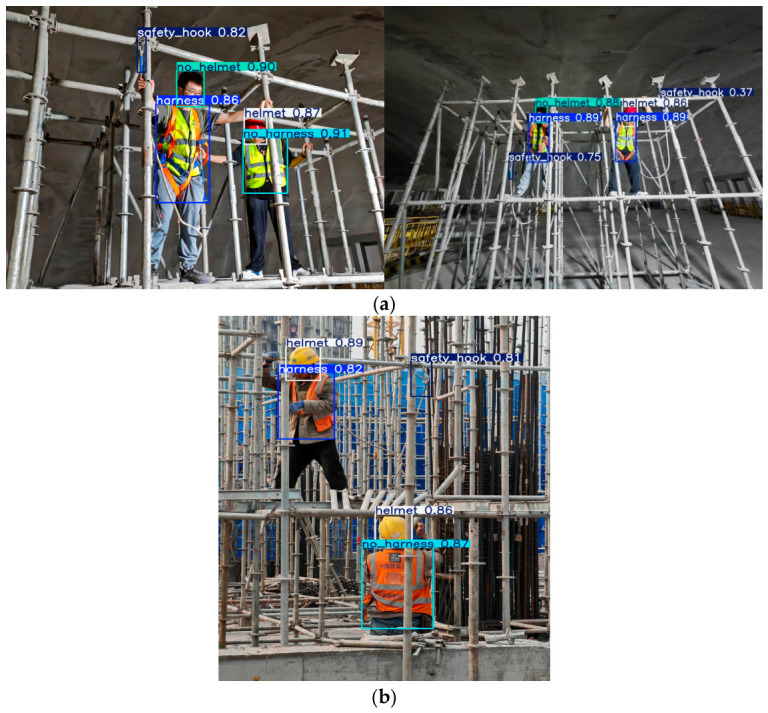
Results of Safety Equipment Detection. (**a**) Results of Experimental Dataset. (**b**) Results of Construction Site Dataset.

Detection results produced by YOLO-ILB on HFSS worker safety equipment are shown in Figure 11. Each output image consists of category labels and bounding boxes. Category labels report the predicted class and confidence score across five types: helmet, no-helmet, harness, no-harness, and safety hook. Unlike helmet and harness, which are body-worn items with a visually distinct unworn state that supports a symmetric presence/absence category pair, the safety hook is a small, detachable component whose absence corresponds to the unannotated background rather than to a visually distinct negative class; a “no-hook” label was therefore not defined, and hook absence is instead handled directly by the compliance recognition logic in Section 3.3, where an image region with no detected hook is treated as a candidate unclipped or excessively distant case. Bounding boxes describe the position and dimensions of each detected target, reflecting the model’s localization capability across equipment categories. From the detection output, compliance with helmet and harness wearing requirements can be assessed directly, while simultaneously detected hook targets provide the spatial data needed for subsequent high-anchoring state recognition.

**Figure 12:**
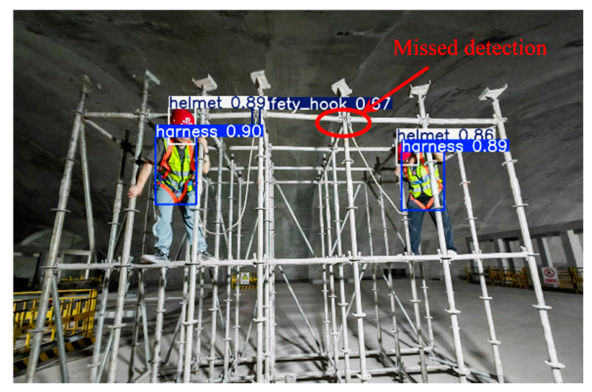
Missed Detection of A Safety Hook.

Failure analysis identifies three primary categories of detection error. First, occlusion-induced failures: partial occlusion of safety hooks by scaffold members removes local feature support, reducing detection confidence below the threshold. Second, appearance-confusion failures: the high visual similarity between safety hooks and scaffold connectors in color and texture makes discrimination difficult at long capture distances; while C3k2_IDWC strengthens small-target feature extraction, edge features and fine-grained local details of hooks captured at extended range or partially overlapping scaffold members are still suppressed by background noise, preventing precise boundary delineation. Third, centroid-displacement failures (occurring at the compliance recognition stage): significant worker bending or rotation causes the harness bounding box centroid to shift, introducing errors into the coordinate-based high/low-anchoring judgment. All three failure types are closely associated with the small-target nature of safety hooks and represent the primary directions for future model improvement. Owing to the model’s strong overall feature extraction capability, a final mAP50 of 0.939 is maintained, satisfying the recognition requirements for all safety equipment categories. An example of missed detection of a safety hook are visualized in Figure 12.

### 4.2. Ablation Experiment

Introducing a single improvement module can enhance model performance to some extent, but the combined effect of multiple modules is not necessarily additive. Interactions between modules may produce synergistic gains or, in some cases, performance degradation. An ablation experiment was therefore designed to isolate the individual contribution of each module and to examine the joint effects of different module combinations. Results are presented in Table 5.

The baseline YOLO11n achieves a mAP50 of 0.910, with 2.583 M parameters, 6.3 G FLOPs, and an inference speed of 256.3 img/s. While detection accuracy and runtime efficiency are both satisfactory, the 0.910 mAP50 leaves room for improvement given the reliability demands of HFSS safety equipment detection. Parameter reduction also remains a potential optimization target.

Each module, when introduced individually, produces a measurable improvement in mAP50, confirming the effectiveness of all three proposed strategies. Adding C3k2_IDWC raises mAP50 to 0.916 while reducing parameters to 2.400 M and increasing inference speed to 270.5 img/s. The parallel decomposition strategy of large-kernel depthwise convolution preserves the wide-range information capture of large kernels, enhances feature extraction, and reduces memory access overhead. Adding SPPF_LSKA alone raises mAP50 to 0.919. The marginal FLOPs increase from SPPF_LSKA (6.3 G to 6.5 G) is attributable to its decomposed horizontal, vertical, and dilated convolution branches, while the subsequent BiFPN replacement removes low-contribution single-input nodes and simplifies cross-scale connections, reducing FLOPs to 5.7 G in the complete model—a net decrease below the baseline as the BiFPN savings more than offset the SPPF_LSKA overhead. Although the large-kernel attention mechanism slightly increases parameters to 2.856 M, inference speed is maintained at 265.6 img/s due to the module’s efficient computation. This confirms that LSKA improves recognition of occluded and small safety equipment targets by enlarging the receptive field and capturing global context more precisely. Replacing PAN with BiFPN produces the largest single-module parameter reduction, cutting parameters by 29% to 1.834 M, while raising mAP50 to 0.922. Bidirectional weighted feature fusion effectively filters redundant channel information and strengthens cross-scale feature interaction, substantially compressing model size while enhancing feature representation.

As modules are added sequentially from YOLO11n-I to the full YOLO-ILB model, mAP50 increases steadily, indicating that the three modules are mutually compatible and complementary in feature space. The complete YOLO-ILB model achieves the best overall performance, with mAP50 raising to 0.939, representing a 3.2% improvement over the baseline. The parameter increase introduced by SPPF_LSKA is offset by the substantial reduction achieved through BiFPN’s streamlined bidirectional fusion architecture, resulting in an overall parameter reduction in the complete YOLO-ILB model. Parameters are reduced to 1.923 M, equivalent to 74.4% of the baseline, while inference speed is maintained at 262.3 img/s. YOLO-ILB achieves a well-balanced trade-off among accuracy, efficiency, and compactness.

### 4.3. Results and Analysis of Safety Harness High-Anchoring Recognition

**Figure 13:**
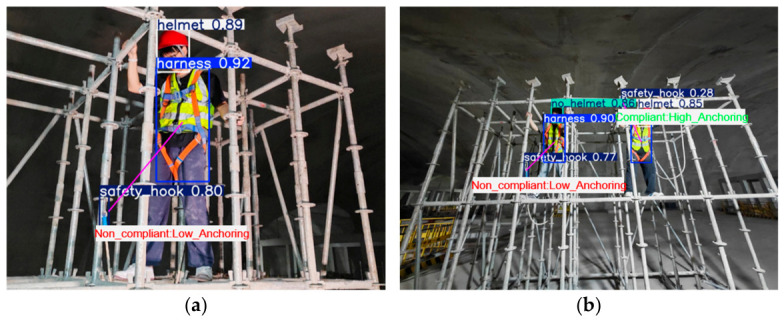
Recognition of high anchoring and low use of safety harnesses. (**a**) Single-target harness usage recognition. (**b**) Multi-target harness usage recognition.

To evaluate the proposed high-anchoring recognition method under real-world conditions, 200 images from the test dataset containing complete detections of workers and their safety equipment were selected for assessment. The 200 images contain a total of 305 validation targets, manually verified and categorized as 138 Compliant High-anchoring cases, 94 Non-compliant Low-Anchoring cases, and 73 dangerous unclipped or excessively distant hook cases. Recognition results are reported in Table 6, with representative examples shown in Figure 13.

The results indicate that the high-anchoring recognition method performs consistently well across all three harness usage states. Individual accuracy rates are 89.86% for High-Anchoring, 90.43% for Low-Anchoring, and 93.15% for Unclipped or Hook Too Far, yielding an overall accuracy of 90.82%. The slightly lower accuracy for High-Anchoring and Low-Anchoring cases is primarily attributable to the reliance on the relative spatial positions of the hook and harness detection boxes. These judgments are susceptible to hook overlap and variation in camera viewpoint. Specifically, large UAV pitch angles introduce perspective distortion in the apparent vertical coordinate relationship; significant lateral camera offsets or rearward worker orientation further cause centroid displacement of the harness bounding box. Importantly, the reported 90.82% overall accuracy was achieved on 305 real field instances that inherently encompass these viewpoint variations, providing empirical support for the practical robustness of the method under real-world deployment conditions. For the unclipped or hook too far category, recognition does not require coordinate-based comparison. When no hook is detected in the image, or when a detected hook fails the lanyard length constraint or the target association matching check, the result is output directly. This simpler decision pathway accounts for the comparatively higher accuracy in this category. Overall, the method reliably identifies different harness usage states in HFSS work scenarios and demonstrates strong recognition performance across all categories.

## 5. Conclusions

This paper presents YOLO-ILB, a task-specific object detector for intelligent PPE monitoring on HFSS, combined with a geometry-based harness anchor point compliance recognition method. The main conclusions are as follows.

Three targeted architectural improvements to YOLO11n produce consistent and substantial performance gains. C3k2_IDWC replaces standard convolutions with multi-branch Inception depthwise convolutions, improving small-target feature discrimination while reducing parameters to 2.40 M and raising inference speed to 270.5 FPS. SPPF_LSKA embeds large separable kernel attention into the SPPF feature aggregation path, enhancing global context awareness at the backbone tail without sacrificing throughput, raising mAP50 to 0.919. BiFPN neck replacement achieves the largest single-module parameter reduction and raises mAP50 to 0.922, demonstrating that learnable bidirectional fusion effectively filters redundant channel information while strengthening cross-scale feature interaction. The complete YOLO-ILB model achieves mAP50 = 0.939 with 1.923 M parameters and an inference speed of 262.3 FPS, establishing a well-balanced Pareto optimum among accuracy, model size, and real-time performance.

Comparative experiments against eight mainstream YOLO detectors confirm that YOLO-ILB outperforms all baselines on the constructed HFSS PPE dataset. It surpasses the second-ranked YOLO11s by 2.8 percentage points in mAP50 while reducing parameters by 79.6% and increasing inference speed by 64.8%, demonstrating that task-specific architectural optimization is more effective than simply scaling model capacity.

The geometry-constrained harness compliance recognition method achieves an overall accuracy of 90.82% across 305 field instances, covering three usage states: compliant high anchoring at 89.86%, non-compliant low anchoring at 90.43%, and dangerous unclipped or excessively distant hook at 93.15%. By combining lanyard validity constraints with minimum-distance hook-to-worker assignment, the method extends safety monitoring from binary PPE presence detection to regulatory compliance assessment, addressing a critical gap in existing construction safety vision systems.

The integrated YOLO-ILB framework supports real-time deployment on resource-constrained hardware, serving both drone-based inspection and fixed-camera continuous monitoring on HFSS construction sites.

Future work will focus on three directions. First, camera pose estimation will be incorporated to compensate for drone tilt angles that may invert the apparent vertical order of hook and harness centroids, reducing errors in anchor point compliance assessment. Second, self-calibrating lanyard length constraint parameters will be developed to generalize across different harness specifications without manual tuning. Third, the pipeline will be extended to multi-frame video streams with temporal tracking, enabling continuous compliance monitoring and automated alert generation. Additionally, a larger dataset with fine-grained annotations of lighting conditions, occlusion levels, and construction-site types will be constructed to support systematic quantitative evaluation of model generalization across sub-conditions.

## Figures and Tables

**Table 1:** Technical parameters of DJI Mini 3 Pro.

Category	Specification	Parameter
UAV	Takeoff weight	<249 g
	Dimensions (L × W × H)	251 × 362 × 70 mm
	Max flight time	47 min
	Max wind resistance	10.7 m/s
	Max tilt angle	40° (forward)/35° (backward)
Camera	Image sensor	1/1.3 inch, 48 MP
	Focal length (equiv.)	24 mm
	Aperture	f/1.7
	Field of view	82.1°
	Shutter speed	1/8000 s–2 s
	Image format	JPEG/DNG (RAW)
Gimbal	Tilt range	−90° to +60°
	Max control speed	100°/s

All models were trained and evaluated on a Windows 11 system equipped with an Intel i5-13600KF processor, 32 GB of RAM, and an NVIDIA RTX 4060 GPU. The software environment comprised Python 3.12 and PyTorch 2.2.2 with CUDA 12.1 and cuDNN 8.8.1.

**Table 2:** Comparative analysis of recent YOLO framework optimizations.

Version	Base	Key Innovations
YOLOv8	YOLOv5	C2f module; optimized loss function
YOLOv10	YOLOv8	Dual-label assignment; PSA module; C2fCIB
YOLO11	YOLOv8	Dual-label assignment; C2PSA; C3k2 module
YOLO12	YOLOv8	Dual-label assignment; A2C2f + C3k2 modules

**Table 3:** Comparison results with different baseline models.

Model	mAP50	Params (M)	FLOPs (G)	FPS (img/s)
YOLOv8n	0.906	2.685	6.8	281.2
YOLOv8s	0.908	9.830	23.4	177.3
YOLOv9t	0.901	1.731	6.4	249.9
YOLOv9s	0.909	6.196	22.1	164.9
YOLOv10n	0.899	2.266	6.5	281.4
YOLOv10s	0.902	7.220	21.4	167.1
YOLO11n	0.910	2.583	6.3	256.3
YOLO11s	0.911	9.415	21.3	159.2
YOLO-ILB	0.939	1.923	5.7	262.3

**Table 4:** Per-category detection performance of YOLO-ILB on the HFSS PPE dataset.

Category	Precision	Recall	AP50
helmet	0.961	0.955	0.975
no-helmet	0.934	0.929	0.949
harness	0.956	0.950	0.968
no-harness	0.925	0.921	0.952
safety hook	0.884	0.846	0.851
Mean	0.932	0.920	0.939

**Table 5:** Comparison of ablation experiments for the YOLO-ILB model. (√: applied; ×: not applied).

Model	C3k2_IDWC	SPPF_LSKA	BiFPN	mAP50	Params (M)	FLOPs (G)	FPS (img/s)
YOLO11n	×	×	×	0.910	2.583	6.3	256.3
YOLO11n-I	√	×	×	0.916	2.400	6.1	270.5
YOLO11n-L	×	√	×	0.919	2.856	6.5	265.6
YOLO11n-B	×	×	√	0.922	1.834	5.7	268.9
YOLO11n-IL	√	√	×	0.929	2.673	6.3	263.0
YOLO-ILB	√	√	√	0.939	1.923	5.7	262.3

**Table 6:** Recognition accuracy of safety harness anchoring compliance across three usage states.

Recognition Type	Sample Size	Correctly Recognized	Individual Accuracy (%)	Overall Accuracy (%)
High-Anchoring	138	124	89.86	90.82
Low-Anchoring	94	85	90.43	
Unclipped/Hook Too Far	73	68	93.15	

## Data Availability

The data used to support the findings of this study are available from the corresponding author upon request.
